# Reproductive Biology of the Sea Cucumber *Holothuria mammata* (Echinodermata: Holothuroidea)

**DOI:** 10.3390/biology11050622

**Published:** 2022-04-19

**Authors:** Eliana Venâncio, Pedro M. Félix, Ana C. Brito, Francisco Azevedo e Silva, Tomás Simões, João Sousa, Susana Mendes, Ana Pombo

**Affiliations:** 1 MARE—Marine and Environmental Sciences Centre, ESTM, Polytechnic of Leiria, 2520-630 Peniche, Portugal; joao.t.sousa@ipleiria.pt (J.S.); susana.mendes@ipleiria.pt (S.M.); ana.pombo@ipleiria.pt (A.P.); 2 MARE—Marine and Environmental Sciences Centre, Faculty of Sciences, University of Lisbon, 1749-016 Lisbon, Portugal; pmfelix@fc.ul.pt (P.M.F.); acbrito@fc.ul.pt (A.C.B.); fhsilva@fc.ul.pt (F.A.e.S.); tfsimoes@fc.ul.pt (T.S.); 3 Departamento de Biologia Animal, Faculty of Sciences, University of Lisbon, 1749-016 Lisbon, Portugal; 4 Departamento de Biologia Vegetal, Faculty of Sciences, University of Lisbon, 1749-016 Lisbon, Portugal

**Keywords:** gametogenesis, gonad development stages, first maturity, gonadosomatic index, NE-Atlantic

## Abstract

**Simple Summary:**

*Holothuria mammata* is one of the sea cucumber species with high commercial value and great demand in Asian markets; however, the existent knowledge about the biology and ecology of this species is scarce. This study aims to describe the reproductive cycle of *H. mammata* in a coastal area of southwest Portugal through monthly collections of biometric data and environmental data. The sex ratio of *H. mammata* was 1:1.2 (male:female), and the size at first sexual maturity was 142 mm for males and 167 mm for females. Gonad development began when days had a shorter photoperiod and lower seawater temperature, and spawning occurred later, with longer photoperiod and higher seawater temperature and chlorophyll-a concentrations. This study is essential to increase the biological and ecological knowledge of the populations of *H. mammata*, create conditions for the domestication of broodstock in captivity and create precise measures for the conservation of ecosystems.

**Abstract:**

*Holothuria mammata* is one of the most valuable species of sea cucumber, as well as one of the main target species harvested in the Mediterranean and NE-Atlantic regions. This study aims to describe the reproductive cycle of *H. mammata* in a coastal area of southwest Portugal. Monthly samplings were carried out for 19 months, with the concomitant collection of environmental data and biometric data. *H. mammata* had a sex ratio of 1:1.2 (male:female) and a size at first maturity of 142 mm for males and 167 mm for females. The gonadosomatic index (GI) peaked between April and May for both sexes. Gonad development started when days had a shorter photoperiod (9 to 13 h of sunlight) and lower seawater temperature (<15 °C), and spawning occurred later, with longer photoperiod (13 to 15 h of sunlight) and higher seawater temperature (>15 °C) and chlorophyll-a concentrations. The development of new studies to increase the biological and ecological knowledge of the populations of *H. mammata* is essential to create conditions for the domestication of broodstock in captivity, to allow the development of fishing regulations based on informed decisions and to create precise measures for the conservation of ecosystems.

## 1. Introduction

Sea cucumbers are benthic echinoderms with elongated bodies, found in virtually all marine environments, from the intertidal zone to the deep sea. These species are mainly marketed as a dry product called *bêche-de-mer* and are considered a traditional delicacy appreciated by Asian consumers due to their nutritional and medicinal properties [1,2,3]. The price normally varies between EUR 43 and EUR 348 per kilogram of dry weight; however, some high-quality species (for example, *Holothuria (Metriatyla) scabra* Jaeger, 1833) can exceed USD 1800 (EUR 1593) per kilogram [4]. The growing demand for this seafood product has led to overexploitation of natural stocks, which are under intense fishing pressure in many parts of the world and require effective conservation measures [5]. Currently, more than 70 species are intensively exploited, mainly in tropical countries, leading to a decline of wild populations [4,6] and, consequently, increasing the demand for this resource in other regions such as the Mediterranean and the NE-Atlantic. In Turkey, catches and exports to the Asian market increased rapidly from 270 tons in 2015 to 1290 tons in 2019 [7]. Most commercial species of holothurian are deposit feeders, feeding on the organic content of sand, mud, and biofilms. Sea cucumbers are important ecosystem engineers who play a fundamental role in the dynamics of the seabed [8,9,10,11], mixing sediments, recycling nutrients, stimulating the growth of algae, and contributing to regulating the carbonate content and pH of the water [12,13,14]. Considering the ecosystem services provided by these species, the overexploitation of multiple populations has the potential to create severe cascading effects in the ecosystems.

*Holothuria mammata* Grube, 1840 is one of the main target species harvested in the Mediterranean, mainly in Turkey, Italy, and Greece, for exportation to Asian markets [7,15,16,17]. Thus, knowledge of biological traits, largely unknown for this group of echinoderms in these regions, is crucial to establishing management and conservation measures for ecosystems, establishing catch limits in fisheries and creating effective methods of aquaculture production for commercial and restocking purposes.

The frequency of reproductive cycles in sea cucumbers is often associated with environmental factors, such as temperature and photoperiod [18,19,20], lunar cycle [21,22], phytoplankton blooms [23,24] and diffusible chemical signals [25], and possibly linked to the specificity of food availability and tidal conditions [26]. On the coast of Peniche (Portugal), *H. mammata* showed an annual reproductive cycle with an increase in gonadosomatic index (GI) from January to April, followed by a decrease in May and a steady increase in June [27]. In another study in southern Portugal (Algarve coast), *H. mammata* demonstrated an annual reproductive cycle with an increase in GI from spring to summer and a drastic decrease in GI from summer to autumn [26]. The reproductive cycle of *H. mammata* differs between regions, being influenced by endogenous and exogenous factors (such as temperature, photoperiod, salinity and food availability). Increasing the spatial range of these studies and understanding the variation in reproductive biology is essential to unraveling the influence of these factors on the gametogenic cycle of *H. mammata*. Likewise, it is critical to determine size at first sexual maturity to impose minimum catch limits in fisheries and in maintenance and reproduction in captivity.

This study aims to overcome the lack of knowledge about the reproductive biology of *H. mammata* on the Portuguese west coast, describing the reproductive cycle for 19 months. The main objectives of this work was: (1) to determine the GI and correlate it with environmental parameters (such as seawater temperature, photoperiod and chlorophyll-a concentration); (2) to determine the stages of gametogenic development (histological analysis of the gonads was performed monthly from January 2018 to July 2019); (3) to provide a detailed description of the reproductive cycle and structures (measurements of the oocyte diameter and thickness of the gonad wall were also obtained and related with the gonad maturation stages); (4) to determine size at first sexual maturity.

## 2. Materials and Methods

### 2.1. Sampling Strategy

About thirty individuals were collected monthly in the NE-Atlantic coastal area, in the Arrábida Marine Park (38°26′50.4″ N; 9°01′58.7″ W), in southwest Portugal, by scuba diving. The collection of individuals started in January 2018 and ended in July 2019. Sea cucumbers were individualized in plastic bags to prevent loss of biological material due to evisceration. All individuals were transported to the laboratory, where they were processed after immersion on ice to promote muscle relaxation and reduce measurements bias [28]. Measurements of total fresh weight (g ± 0.01) and total length (mm ± 1) were recorded. Subsequently, an incision was made along the ventral surface to remove the organs, including gonads; the gutted weight (g ± 0.01) and gonad wet weight (g ± 0.01) were also recorded. The GI was estimated according to Benítez-Villalobos et al., 2013 [29]:

(1) $$\mathrm{GI}=\frac{gonad\;weight}{gutted\;weight}\times 100$$

The environmental parameters were obtained from January 2018 to March 2019. Seawater temperature and chlorophyll-a concentration were collected once a month after measurement stabilization using a multiparametric sonde YSI-EXO2 (Yellow Springs, OH, USA). Photoperiod was also registered, based on the NAV Portugal E.P.E. records (www.nav.pt/en/ais/sunrise-sunset-tables, accessed on 20 December 2019). These variables were used to identify any potential relationship with gonad maturation and assess the influence of these factors on the reproductive cycle of *H. mammata*.

### 2.2. Histological Analysis

The gonads were immediately fixed in a buffered 4% formalin solution for 48 h to perform the histological study. The samples were then processed in a Leica^®^ TP1020 Automatic Tissue Processor (Leica Microsystems GmbH, Wetzlar, Germany) with sequential submersions in graded ethanol for dehydration followed by xylene for clarification and impregnation with paraffin wax at 60 °C. After the gonad samples were embedded in 100% (*v*/*v*) paraffin, they were cut with a thickness of 7 µm using an Accu-Cut^®^ SRM™ 200 Rotary Microtome (Sakura Finetek Europe BV, Alphen aan den Rijn, The Netherlands) and stained with Harris’ haematoxylin solution (Scharlab S.L., Sentmenat, Barcelona, Spain) and eosin Y (yellowish) (VWR International, Leuven, Belgium). Gonad tissues were then analyzed using a Leica^®^ DM 2000 LED light optical microscope equipped with a Leica^®^ MC170 5MP HD Microscope Camera and the combined LAS v4.4.0 software (Leica Application Suite) for monitor display (Leica Microsystems GmbH, Wetzlar, Germany). Only a section of a gonadal tubule was used to obtain the definitive preparations. Gametogenesis was classified into five stages of gonad development, according to previous studies [30,31,32]. Measurements of oocytes were performed along the major axis and only those whose nucleus was visible. The diameter of 30 oocytes (randomly and per female) was recorded in all females each month. In order to determine the maturation stages, the entire histological preparation was analyzed. The spawning stage was verified through microscopic observations of the gonads, gaps in the lumen, the wrinkled gonad wall and the presence of phagocyte clusters were detected. The gonad wall was analyzed in all sampled individuals. For each individual, the average thickness was determined by measuring the thickness at 10 different points. The monthly mean thickness was presented as mean ± SD.

### 2.3. Data Analysis

The sex ratio (male:female) was calculated monthly, and the deviation from the expected ratio (1:1) was tested by the chi-square adjustment test (χ2). Differences in monthly GI between sexes were tested using the parametric *t*-test (in which all assumptions associated with normality and homogeneity of variances were validated). The association between gonad developmental stages and months was tested using the chi-square test for independence (χ2). All requirements were validated. However, when these were not met, the analysis was performed using suitable tests in order to obtain accurate results [33,34]. The size at maturity was estimated based on the size at which 50% of all individuals have the probability of being mature, with a logistic regression based on a frequentist general linear model (GLM) and a non-parametric bootstrap method with 999 iterations.

The cross-correlation tests were performed in order to determine the relationships between the reproductive cycle and external factors, in which the gonadosomatic index was analyzed as a function of temperature, chlorophyll-a concentration and photoperiod. Several time lags were considered, and correlations were statistically significant at a level of 5% (i.e., when *p*-value < 0.05). Gonadal wall thickness was compared between sexes by maturation stage using a one-way analysis of variance (ANOVA). Moreover, differences in oocyte diameter by month were analyzed using one-way ANOVA. When the data failed to meet the assumptions (that is, normality of data and homogeneity of variances), the Kruskal–Wallis non-parametric test was used. Whenever applicable, the Games–Howell multiple comparisons tests were carried out according to the fulfillment or not of the variance analysis assumptions.

Statistical treatment was performed using the IBM SPSS Statistics v27.0 software and R software 2013 [35] with package sizeMat v1.1.2 [36]. When applicable, values were presented as mean ± standard deviation (SD). All differences were considered statistically significant at the significance level of 5% (that is, *p*-value < 0.05).

## 3. Results

### 3.1. Sex Ratio

**Figure 1:**
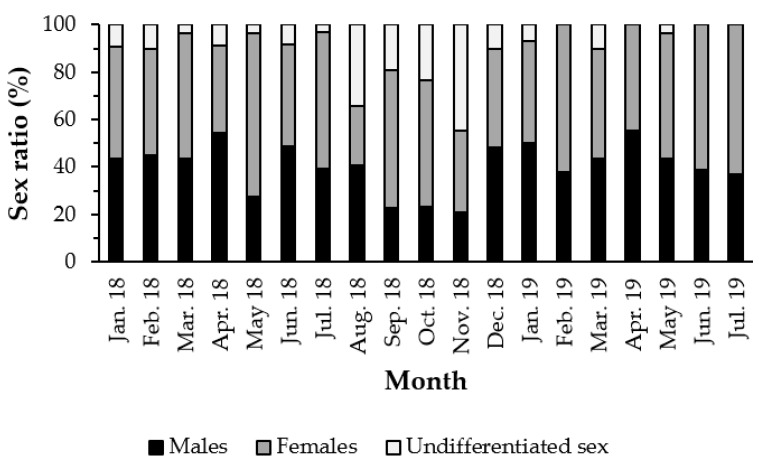
Sex ratio (%) separated by sex and month, representative of the population of *Holothuria mammata* in the coastal area of the NE-Atlantic in southwest Portugal.

The study of the reproductive cycle of *H. mammata* was determined considering the microscopic observation of the gonads of 585 adult individuals, for 19 months, with an average length of 227.44 ± 45.39 mm and an average weight of 345.20 ± 124.30 g. In total, 236 males (40.34%), 288 females (49.23%) and 61 undetermined (10.43%) were sampled. The sex ratio of *H. mammata* differs significantly from the 1:1 ratio, demonstrating a ratio of 1:1.2 (χ^2^ = 10.09; *p*-value < 0.05), with a higher proportion of females than males in most months (Figure 1).

### 3.2. Gonadosomatic Index

**Figure 2:**
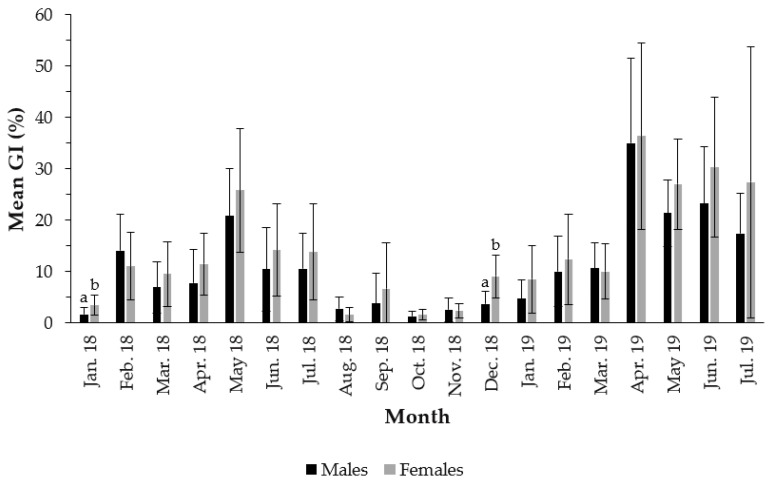
Monthly means (± SD) of the Gonadosomatic index of *Holothuria mammata* in the coastal area of the NE-Atlantic in southwest Portugal. Different letters (a and b) indicate statistical differences between males and females at a significance level of 0.05.

The temporal variation in the GI over the sampling period showed a peak in May 2018 and several peaks in April, May, June and July 2019, for both sexes (Figure 2). In 2018, the average GI reached its peak in May (20.80% for males and 25.85% for females), with no statistically significant differences between males and females (t_(26)_ = 1.19; *p*-value = 0.25). In 2019, several peaks were recorded with no statistically significant differences between males and females; in April (35.02% for males and 36.37% for females, t_(27)_ = 0.21; *p*-value = 0.84), May (21.35% for males and 27.00% for females, t_(27)_ = 1.93; *p*-value = 0.06), June (23.25% for males and 30.32% for females, t_(29)_ = 1.51; *p*-value = 0.14) and July (17.37% for males and 27.40% for females, t_(28)_ = 1.22; *p*-value = 0.23). The mean GI was minimal in October 2018 for both sexes (females = 1.59% and males = 1.31%), with no statistically significant differences (t_(21)_ = 0.64; *p*-value = 0.54). Although there were no differences between the GI of males vs. females in the months when the GI peaked, these differences were evident in January 2018 (t_(27)_ = 2.86; *p*-value < 0.01) and December 2018 (t_(24)_ = 3.91; *p*-value < 0.01).

### 3.3. Reproductive Cycle

**Figure 3:**
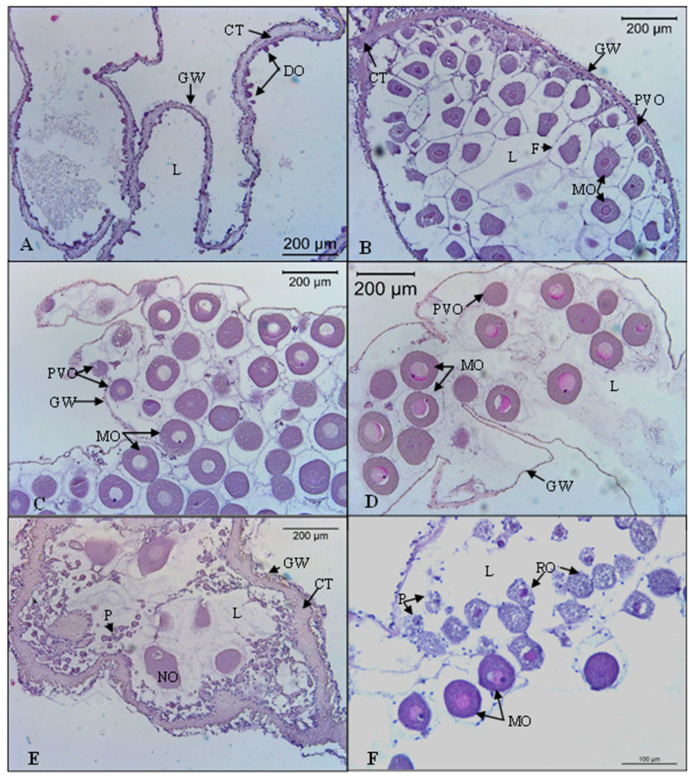
Microscopical characteristics of gonad development stages of the females of *Holothuria mammata*. (**A**) Recovery stage (November 2018 sample); (**B**) Growing stage (March 2019 sample); (**C**) Mature stage (May 2019 sample); (**D**) Spawning stage (June 2019 sample); (**E**,**F**) Post-spawning stage (August 2018 sample). CT: connective tissue; DO: developing oocyte; GW: gonad wall; L: lumen; MO: mature oocyte; NO: Non-spawned oocyte P: phagocytes; PVO: previtellogenic oocyte; RO: relict oocyte.

In females, the recovery stage (I) was characterized by the maximum thickness of the ovary wall. Small developing oocytes arranged in a single layer were observed in the germinal epithelium (Figure 3A). The growing stage (II) was characterized by the ovarian wall still thick, but, over time, it began to narrow. The germinal epithelium had oocytes at various stages of development. As vitellogenesis progressed, fully-grown oocytes began to take up a central position in the lumen. The oocytes were surrounded by small follicular cells (Figure 3B). The mature stage (III) was characterized by the ovary wall with minimal thickness. The lumen was filled with mature oocytes with a well-defined nucleus. In the germinal epithelium, previtellogenic oocytes could be present and continue their development (Figure 3C). The spawning stage (IV) was characterized by the thin ovary wall. A remarkable decrease in the abundance of oocytes within the tubules was observed. The lumen had empty spaces due to the release of gametes. The number of oocytes and the size of the gaps in the lumen depended on the spawning stage. Some previtellogenic oocytes were observed in the germinal epithelium (Figure 3D). The post-spawning stage (V) presented the ovary wall thicker and wrinkled, with a large amount of connective tissue. The lumen presented traces of follicular epithelium and phagocytes. Non-spawned oocytes were significantly degraded due to phagocytic activity (Figure 3E,F).

**Figure 4:**
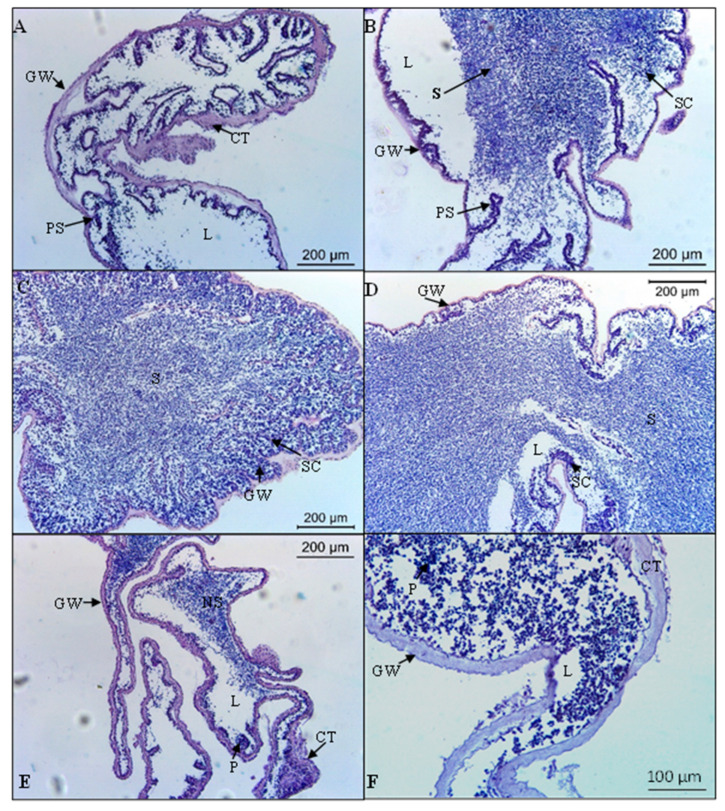
Microscopical characteristics of gonad development stages of the males of *Holothuria mammata*. (**A**) Recovery stage (November 2018 sample); (**B**) Growing stage (April 2019 sample); (**C**) Mature stage (May 2019 sample); (**D**) Spawning stage (June 2019 sample); (**E**,**F**) Post-spawning stage (October 2018 sample). CT: connective tissue; GW: gonad wall; L: lumen; NS: non-spawned spermatozoa; P: phagocytes; PS: primary spermatocytes; S: Spermatozoa; SC: spermatocyte columns.

In males, the recovery stage (I) was characterized by the thickening of the external and internal epithelium of the gonadal wall. The germinal epithelium presented numerous folds that increased the surface area for spermatogenesis and primary spermatocytes (Figure 4A). The growing stage (II) was characterized by the narrowing of the gonad wall, and the folds of the epithelium began to straighten. Columns of spermatocytes extended towards the lumen, and spermatozoa began to fill it (Figure 4B). The mature stage (III) was characterized by a thin gonad wall and the lumen filled with densely packed spermatozoa. Few spermatocytes were present along the germinal epithelium (Figure 4C). The spawning stage (IV) presented thin and wrinkled spawned tubules with empty spaces, which were present in the lumen due to the release of gametes. A thicker spermatogenic column separated from the central part of the lumen (Figure 4D). The post-spawning stage (V) was characterized by thickened and wrinkled gonad wall with a large amount of connective tissue. The lumen presented non-spawned spermatozoa, debris and phagocyte clusters (Figure 4E,F).

**Figure 5:**
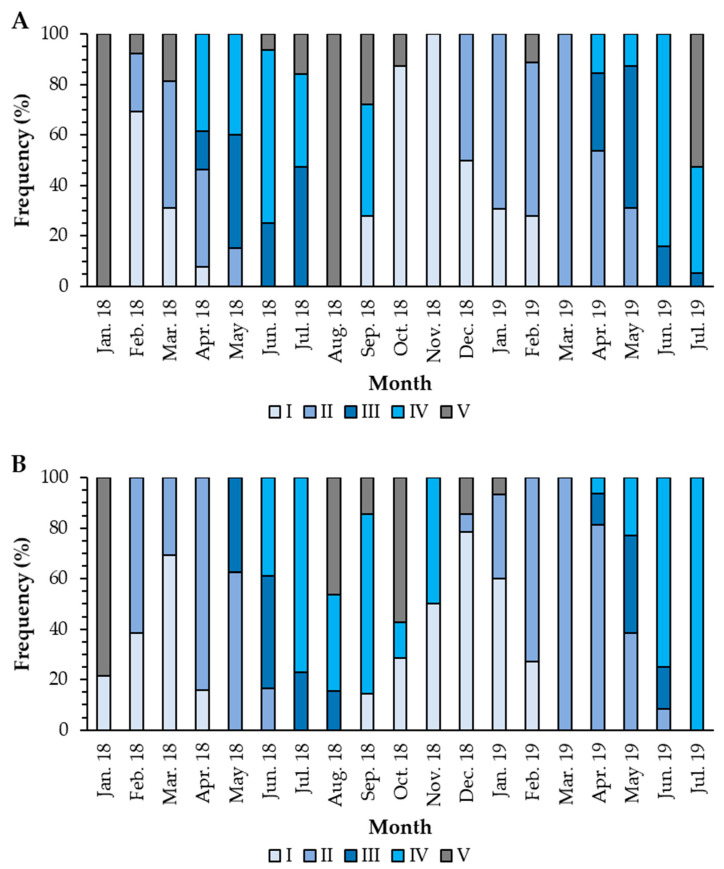
Temporal allocation of gonad development stages in females (**A**) and males (**B**) of *Holothuria mammata* in the coastal area of the NE-Atlantic in southwest Portugal. I—Recovery; II—Growing; III—Mature; IV—Spawning; V—Post-spawning.

The females presented the recovery period from February to April 2018 and from September 2018 to February 2019. The growing stage occurred from February to May 2018 and from December 2018 to May 2019. The mature stage occurred from April to July, both in 2018 and 2019, and the spawning period occurred from April to September 2018 and from April to July 2019 (Figure 5A). In males, the recovery period occurred from January to April 2018 and from October 2018 to February 2019. The growing stage occurred from February to June 2018 and from January to June 2019. Mature gonads were found from May to August 2018 and from April to June 2019. The spawning period occurred from June to November 2018 and from April to July 2019 (Figure 5B). There was a statistically significant association (Fisher’s exact test; *p*-value < 0.001) between the stages of gonad development and the sampling months, demonstrating that the months can explain the stage of maturation in which the individuals are. Although there is some lag between males and females in 2018, the spawning season is more synchronized between June and September. In 2019, spawning was synchronized and occurred between April and July.

#### 3.3.1. Gonadal Wall Thickness

**Figure 6:**
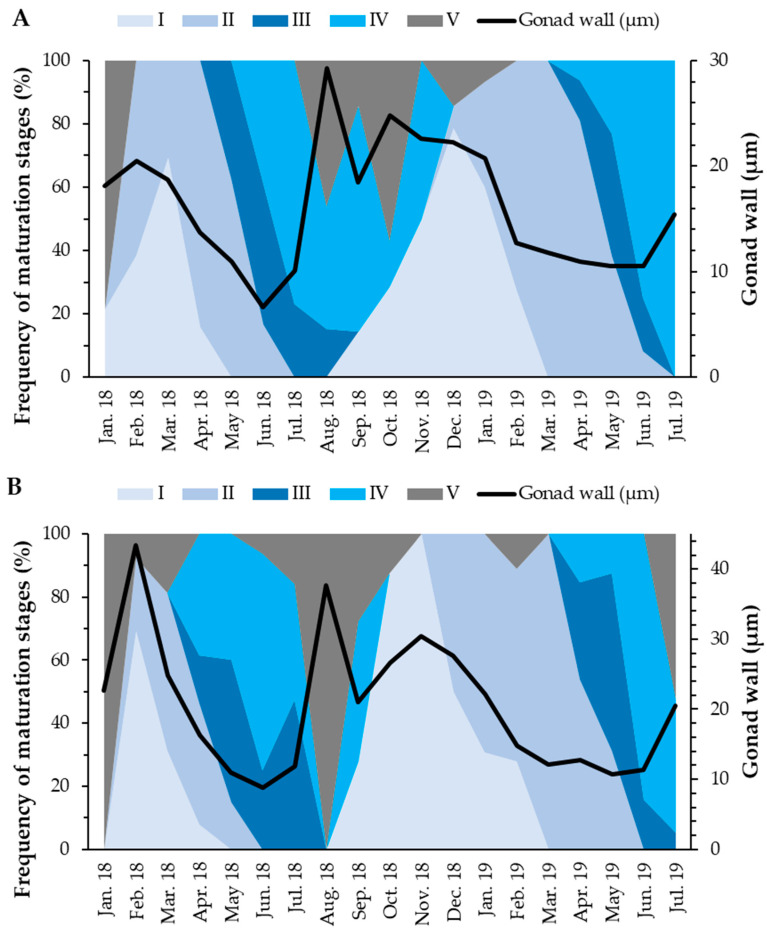
Frequency of maturation stages and average thickness of the gonad wall of males (**A**) and females (**B**) of *Holothuria mammata* in the coastal area of the NE-Atlantic in southwest Portugal. I—Recovery; II—Growing; III—Mature; IV—Spawning; V—Post-spawning.

In females, the gonad wall thickness was maximum in February 2018 (43.36 µm), with two more peaks in August and November 2018 (37.63 and 30.37 µm, respectively). On the other hand, males showed two annual peaks in August 2018 (29.25 µm) and October 2018 (24.76 µm). Both sexes presented statistically significant differences in the thickness of the gonad wall when comparing the maturation stages (Kruskal–Wallis test; *p*-value < 0.001). Females did not show statistically significant differences between gonad wall thickness in stage I and stage V (Games–Howell test; *p*-value = 0.51). On the other hand, males did not show statistically significant differences between the gonad wall thickness in the stages II and IV (Games–Howell test; *p*-value = 0.77) and between the gonad wall thickness in the stages I and V (Games–Howell test; *p*-value = 0.34). In both sexes, the gonad wall tended to be thicker when individuals were in the pos-spawning or recovery stages (Figure 6).

#### 3.3.2. Oocyte Diameter

**Figure 7:**
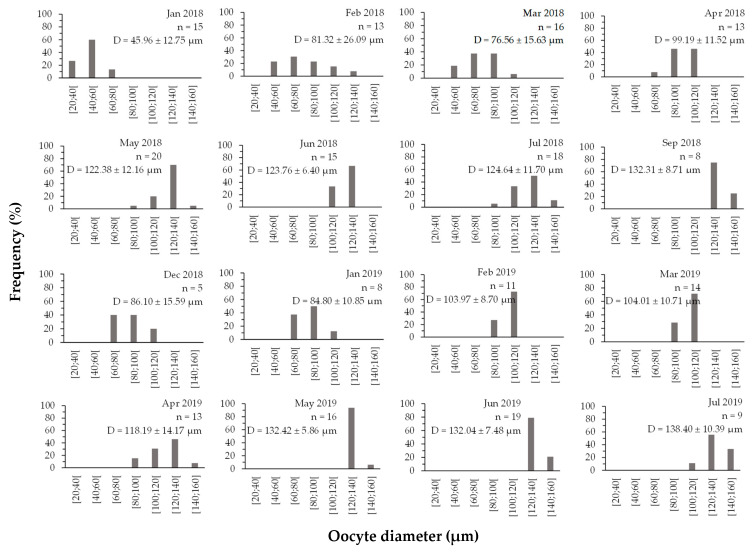
Monthly distribution of oocyte diameter in size classes (January 2018–July 2019) of *Holothuria mammata* in the coastal area of the NE-Atlantic in southwest Portugal. *n* = number of females; D = mean diameter ± SD.

Oocyte diameter progressed as the gonads developed, and the maximum diameter was observed when the gonads were mature and ready to spawn (Figure 7). Primary and secondary (non-vitellogenic, <100 µm) oocytes occurred mainly from January to April and December 2018 and January 2019, coinciding with the period of gonadal growth. On the other hand, vitellogenic oocytes (>120 µm) were more frequent from May to July and September 2018 and from July 2019, when they were ready to spawn. Statistically significant differences are found between April 2019 (118.19 ± 14.75 µm) and May 2019 (132.42 ± 6.05 µm, *p*-value = 0.031), between April 2019 and June 2019 (132.04 ± 7.68 µm, *p*-value = 0.040), between April 2019 and July 2019 (138.40 ± 11.02 µm, *p*-value = 0.019) and between May 2018 and July 2019 (122.38 ± 12.47 µm, *p*-value = 0.040).

### 3.4. Reproductive Cycle vs. Environmental Factors

**Figure 8:**
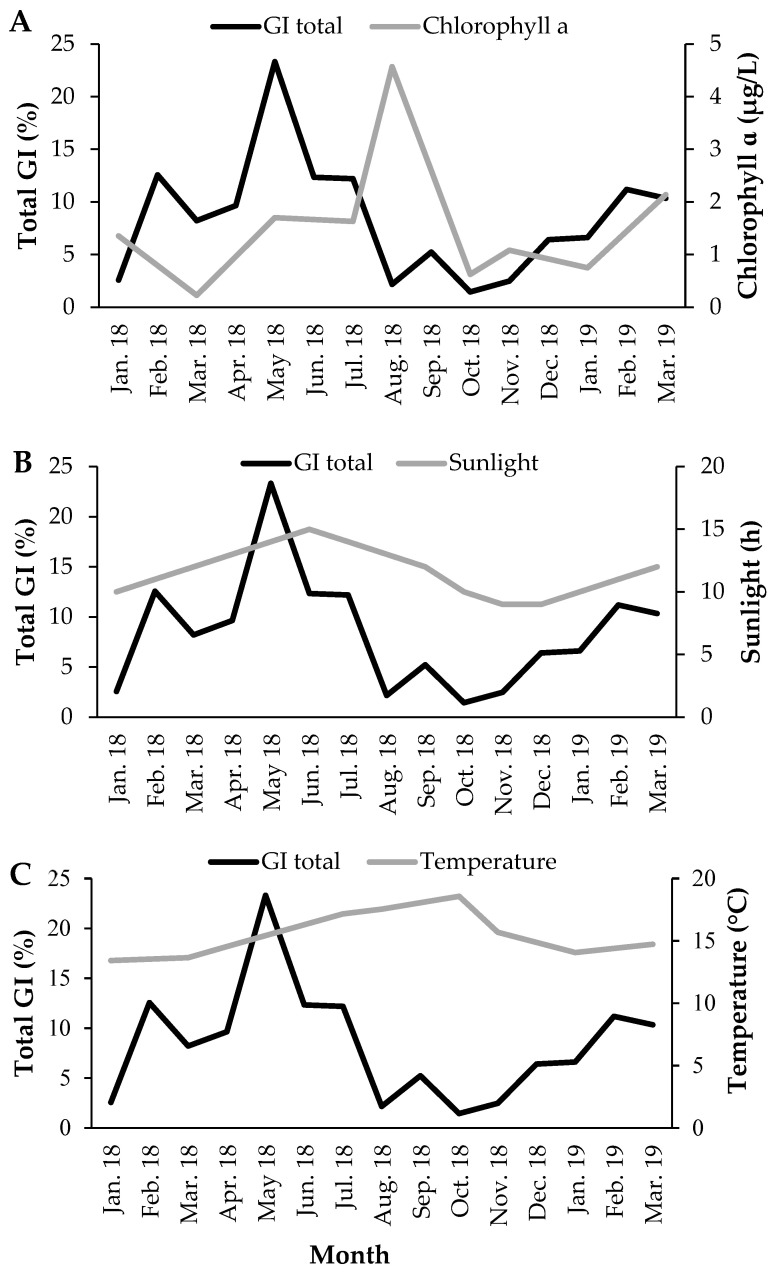
Temporal pattern of environmental factors and Gonadosomatic index of *Holothuria mammata* in the coastal area of the NE-Atlantic in southwest Portugal. (**A**) Chlorophyll-a, (**B**) Photoperiod, (**C**) Seawater temperature.

**Figure 9:**
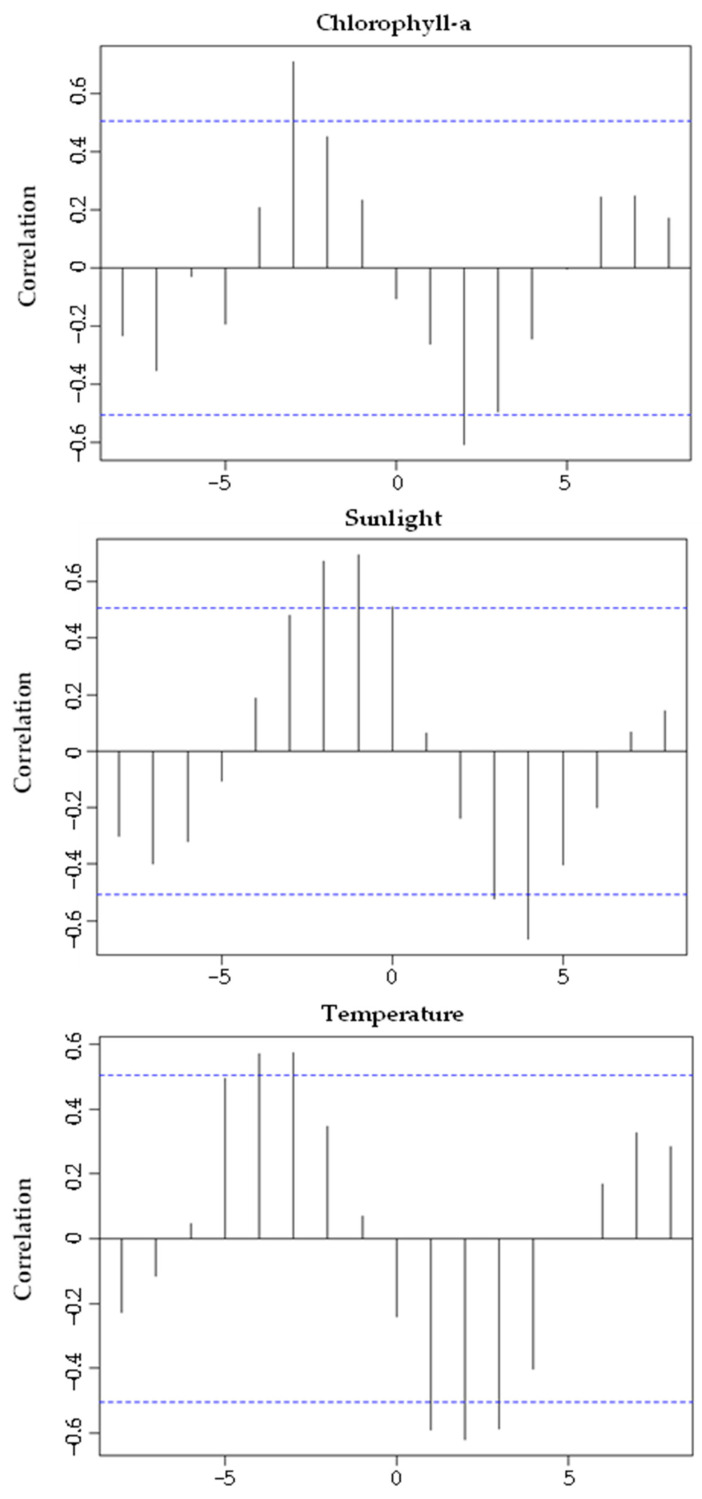
Cross-correlation between the Gonadosomatic index of *Holothuria mammata* and environmental factors, with different time lags. The blue line indicates a significance level of 0.05.

**Figure 10:**
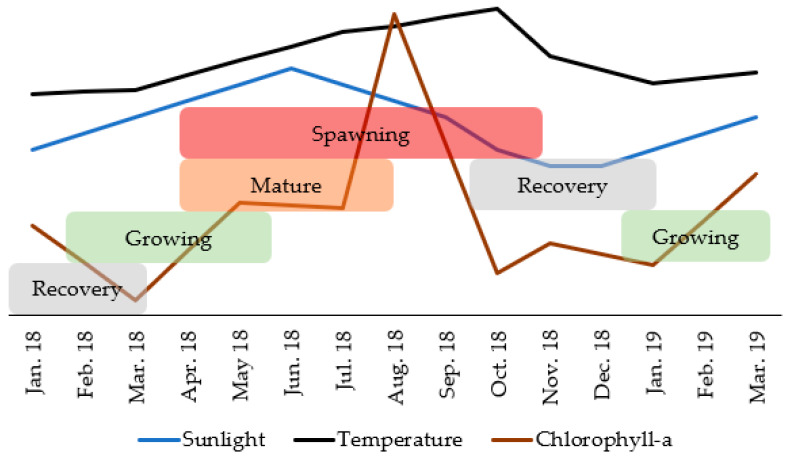
Diagram representing the concomitant evolution of the maturation stages in *Holothuria mammata* and the environmental parameters significantly correlated with the Gonadosomatic Index. Recovery (I); Growing (II); Mature (III); Spawning (IV).

Chlorophyll-a concentration (Figure 8A), photoperiod (Figure 8B) and seawater temperature (Figure 8C) correlated significantly with GI (with a time lag) (Figure 9). The GI presented a positive correlation, with a time lag, with chlorophyll-a (R = 0.608; *p*-value < 0.05), its maximum occurred two months after the GI peak. GI showed a positive correlation (with a time-lag of one month) with the photoperiod (R = 0.766; *p*-value < 0.05), its maximum occurred after the GI peak. The GI and temperature increased together from January to May 2018; however, this trend then changed (from May to October 2018, the GI decreased dramatically, and the temperature continued to rise; from October 2018 to March 2019, the GI increased, and the temperature decreased), a change that reflects a negative correlation (with a time lag) (R = −0.687; *p*-value < 0.05). Thus, the recovery period occurred on days of shorter duration and with lower temperature and concentration of chlorophyll-a. Gametogenesis occurred as the photoperiod, temperature, and chlorophyll-a increased. The spawning period coincided with higher temperature, photoperiod, and chlorophyll-a concentration (Figure 10).

### 3.5. Size at First Sexual Maturity

**Figure 11:**
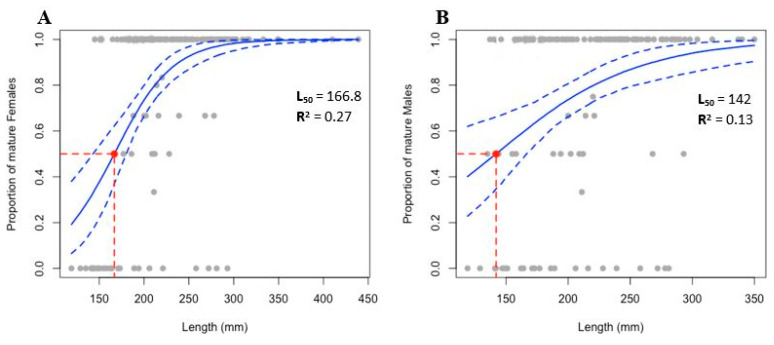
Size at first maturity for (**A**) Females and (**B**) Males of *Holothuria mammata*, where the L_50_ (dashed red line) represents the length at which a randomly chosen specimen has a 50% chance of being mature. The fitted values for the logit regression are based on a frequentist GLM and a non-parametric bootstrap method. The solid blue lines represent the proportion of mature by length. The confidence intervals (95%) are represented by dashed blue lines.

The estimation of size at first maturity revealed an L_50_ of 166.8 mm for females and a lower value for males, which mature at 142 mm (Figure 11). However, the results showed a low representation of immature individuals within the sampled population.

## 4. Discussion

### 4.1. Sex Ratio

The population of *H. mammata* in the Arrábida Marine Park had a sex ratio of 1:1.2 (male:female). In fact, several studies on the reproductive biology of sea cucumbers corroborated the imbalance in the sex ratio. Santos et al., 2017 [27] described a sex ratio of 1:1.3 (male:female) in a population of the same species on the coast of Peniche (Portugal) and, for other sea cucumber species, showed a similar trait; most of which favored females. *Holothuria whitmaei* presented a sex ratio of 1:1.3 (male:female) in the Pacific Ocean [37], *Holothuria leucospilota* showed an unbalanced sex ratio of 1:11.2 (male:female) in the Western Indian Ocean [18], and *Holothuria arenacava* presented a sex ratio of 1:1.2 (male:female) on the Kenyan coast [38]. Previous studies pointed to fishing pressure as a possible cause for the unbalanced sex ratio [27,37,39] due to the tendency that certain species have to cluster in same-sex groups or due to night fishing when individuals are more active, although it is not known whether the nocturnal activity is similar in both sexes [40]. However, this study was carried out in a marine protected area (MPA), where recruitment is successful, and the population is unexploited [41], suggesting this ratio is a natural trait for these detritivorous sea cucumbers.

### 4.2. Reproductive Cycle

Temperate sea cucumber species generally have an annual reproductive pattern with discrete spawning periods in spring and summer [27,30,31,42,43,44]. The population of *H. mammata* considered in this study had an annual reproductive peak with a GI peak in May 2018 and several GI peaks in 2019 (April to July). This is similar to what was previously reported for the coast of Peniche [27], where the GI peak occurred in the spring. Moreover, Navarro et al., 2012 [44] reported that *H. mammata* and *H. sanctori* had maximum GI in warm months in Gran Canaria, Spain (Eastern Atlantic Ocean). These observations may indicate that the reproductive cycles of *H. sanctori* and *H. mammata* may be synchronized, as reported for other echinoderms [27,37,39,45]. On the other hand, a population of *H. mammata* on the southern coast of the Algarve (Portugal) showed the maximum GI in summer followed by an abrupt decrease [26], indicating that reproduction occurred later (summer–autumn).

According to histological analysis, the recovery period occurred from October to January, and gametogenesis occurred from January to May. The gametogenic cycle of *H. mammata* showed a time lag between males and females in 2018, in the mature stage (April–July in females and May–August in males) and in the spawning stage (April–September in females and June–November in males). This delay of males in relation to females can have consequences on fertilization success and consequently on the recruitment of juveniles. However, this event may be related to the fact that each individual has gonad tubules at different stages of maturation (individuals do not expel all mature gametes at once) [46,47], and in this study, only one gonad tubule per individual was analyzed. The females presented some unspawned oocytes, making it difficult to identify the exact stage of maturation. On the other hand, great synchronization between males and females was verified in 2019, with the spawning period between April and July.

Gonadal wall thickness was evaluated for the first time for *H. mammata*. The average thickness of the gonad wall was greater in the recovery and post-spawning stages; during gametogenesis, the gonad wall became thinner, reaching its minimum thickness when it reached its maximum maturation point. As the spawning progresses, the gonad wall becomes thicker until reaching the maximum thickness in the post-spawning stage. Although there are no studies evaluating the influence of the gonad wall thickness on the various stages of gonad maturation, other studies were dedicated to the evaluation of the gonad tubule diameter [27,29,30,31,42,44,45,46,48,49], demonstrating an inverse relationship to gonad wall thickness, because as the lumen is filled, the wall thickness decreases and the diameter of the tubule increases.

The oocyte diameters were distributed in size classes, demonstrating that females had a higher frequency of smaller oocytes (<80 µm) between December and January, when they were in the recovery or post-spawning stages. The growing stage had oocytes with a diameter of up to 100 µm (February to April), mature females had oocytes with 100–120 µm, and spawning occurred from May to September, when the oocytes reached a diameter greater than 120 µm. These results corroborate the study of Santos et al., 2017 [27], who demonstrated that mature oocytes had a diameter between 108 and 122 µm for *H. mammata*. Moreover, Tuwo and Conand, 1992 [42] demonstrated that mature individuals of *H. forskali* had oocytes with a diameter between 90 and 120 µm. Other studies reported that mature oocytes and non-mature oocytes are found at all stages of gonad maturation in other sea cucumber species [30,44,45,46]; however, in this study, no mature oocytes were found in the recovery period, and no immature oocytes were found in the spawning stage.

### 4.3. Reproductive Cycle vs. Environmental Factors

In general, the periodicity of the reproductive cycles of sea cucumbers is associated with environmental factors such as seawater temperature, food availability, and photoperiod [18,19,20,21,24]. The development of the gonads started with the lowest seawater temperature (<14 °C) and days of shorter duration (<12 h of sunlight), and spawning occurred when the photoperiod approached the maximum (>14 h of sunlight) and the seawater temperature peaked (>15 °C) (Figure 10), as reported in previous studies [27,44]. The negative association of GI with temperature was markedly influenced by the continuous rise of temperature while the GI turned to a steep decrease due to gamete release. However, the temperature may contribute to cue gonad maturation as GI rises, in April, shortly after the onset of temperature increase, in March. The temperature may also have an indirect effect on reproduction, increasing food availability through increased benthic productivity [38] and, consequently, favoring larval development [50]. Phytoplankton was evaluated through the concentration of chlorophyll-a, used as a proxy of phytoplankton biomass, which peaked three months after the GI. In other words, the increase in the biomass of microalgae occurred in the summer, when *H. mammata* was spawning. Indeed, a synchronization of spawning according to phytoplankton availability is considered a common advantageous adaptation in marine invertebrates [27,44,51,52]. The energy required for the development of the gonads depends on primary productivity; although *H. mammata* is not a suspension feeder, its diet depends on the organic content of the sediment rich in microalgae. The first larval stages of sea cucumbers are planktonic and planktotrophic. *H. mammata* feeds from the 3rd to the 17th day at 21 °C [11] and from 2nd day to the 12th day at 25–28 °C [53] and needs phytoplankton for their development. The coincidence of the chlorophyll-a peak with reproduction is also described for this same species on the southern coast of Portugal [26] and may be related to the survival of the larval stages and not to gametogenesis. Thus, the success in the reproduction and development of the larvae of *H. mammata* is strongly influenced by the environmental parameters. In Ria Formosa (Algarve, Portugal), the temperature reaches 27 °C in summer [53], and the greater availability of food guarantees a better and faster development of the larvae.

### 4.4. Size at First Sexual Maturity

The size of the first maturity for *H. mammata* showed that this species matures at a smaller size than any other species previously reported, either temperate or tropical [26,44,54,55,56,57,58]. Although with a low representation of immature individuals, cutting off the left logistic tail. This represents the first estimation of L_50_ for *H. mammata* and may be regarded as a preliminary evaluation, requiring additional data for lower size classes. However, the length at first maturity can never be underestimated in the present study. The bias may exist toward smaller individuals that may reach sexual maturity even earlier than the values presented herein.

### 4.5. Implications for Fisheries and Aquaculture Management

Sea cucumbers are caught all over the world to respond to Asian demand. The lack of management measures to regulate these fisheries has led to a decrease in wild populations and the destruction of ecosystems. Information on reproductive biology and size at first sexual maturity is essential to create management measures such as a closed season (allowing mature individuals to reproduce) and minimum catch size [4]. In this study, the spawning period was determined in spring–summer, with a higher incidence between June and September, and the size at first maturity was 167 mm in females and 142 mm in males. In this way, the seasonal ban should take place in the summer, and the catch the minimum catch size should be set to at least 200 mm, above a species’ first size at maturity in order to allow most individuals to spawn at least [59] once and because sea cucumbers have no sexual dimorphism. Our data are also an asset for aquaculture production for commercial, scientific or restocking purposes, providing important biological information for the implementation of effective techniques in maintenance, spawning induction and larval development through temperature and photoperiod manipulation and availability of food [48].

## 5. Conclusions

*H. mammata* presented an annual reproduction pattern with a long spawning period (April to November), with greater incidence and synchronism (between males and females) from June to September. The reproductive period is closely related to environmental parameters, as spawning occurred when days were longer and temperature and chlorophyll-a reached their peak.

Currently, the status of the population of *H. mammata* in Portugal is unknown, but its exploitation is a reality based on apprehensions of illegal landings in national waters. It is essential to acquire biological and ecological knowledge of wild populations to create precise measures for the conservation of these species and, consequently, the benthic ecosystems.

## Data Availability

The datasets generated during and/or analyzed during the current study are available from the corresponding author on reasonable request.

## References

[1] Conand C. (1990). The Fishery Resources of Pacific Island Countries. Part 2: Holothurians.

[2] Purcell S.W., Lovatelli A., Vasconcellos M., Ye Y. (2010). Managing Sea Cucumber Fisheries with an Ecosystem Approach.

[3] Santos R., Dias S., Pinteus S., Silva J., Alves C., Tecelão C., Pedrosa R., Pombo A. (2015). Sea cucumber *Holothuria forskali*, a new resource for aquaculture? Reproductive biology and nutraceutical approach. Aquac. Res..

[4] Purcell S.W., Williamson D.H., Ngaluafe P. (2018). Chinese market prices of beche-de-mer: Implications for fisheries and aquaculture. Mar. Policy.

[5] Toral-Granda V., Lovatelli A., Vasconcellos M. (2008). Sea Cucumbers—A Global Review of Fisheries and Trade.

[6] Meloni D., Esposito G. (2018). Hygienic and commercial issues related to the illegal fishing and processing of sea cucumbers in the Mediterranean: A case study on over-exploitation in Italy between 2015 and 2017. Reg. Stud. Mar. Sci..

[7] Derelї H., Aydın M. (2021). Sea cucumber fishery in Turkey: Management regulations and their efficiency. Reg. Stud. Mar. Sci..

[8] Sonnenholzner J. (2003). Seasonal variation in the food composition of *Holothuria theeli* (Holothuroidea: Aspidochirotida) with ob-servations on density and distribution patterns at the central coast of Ecuador. Bull. Mar. Sci..

[9] MacTavish T., Stenton-Dozey J., Vopel K., Savage C. (2012). Deposit-Feeding Sea Cucumbers Enhance Mineralization and Nutrient Cycling in Organically-Enriched Coastal Sediments. PLoS ONE.

[10] Durden J.M., Schoening T., Althaus F., Friedman A., Garcia R., Glover A.G., Greinert J., Stout N.J., Jones D.O., Jordt A., Hughes R.N., Hughes D.J., Smith I.P., Dale A.C. (2016). Perspectives in visual imaging for marine biology and ecology: From acquisition to understanding. Oceanography and Marine Biology: An Annual Review.

[11] Venâncio E., Félix P.M., Brito A.C., Sousa J., Azevedo e Silva F., Simões T., Narciso L., Amorim A., Dâmaso L., Pombo A. (2021). Do broodstock diets influence viability and larval development of *Holothuria mammata*?. Aquaculture.

[12] Uthicke S. (2001). Interactions between sediment-feeders and microalgae on coral reefs: Grazing losses versus production enhancement. Mar. Ecol. Prog. Ser..

[13] Uthicke S. (2001). Nutrient regeneration by abundant coral reef holothurians. J. Exp. Mar. Biol. Ecol..

[14] Purcell S.W., Polidoro B.A., Hamel J.-F., Gamboa R.U., Mercier A. (2014). The cost of being valuable: Predictors of extinction risk in marine invertebrates exploited as luxury seafood. Proc. R. Soc. B.

[15] Aydin M. (2008). The commercial sea cucumber fishery in Turkey. SPC Beche-De-Mer Inf. Bull..

[16] González-Wangüemert M., Aydin M., Conand C. (2014). Assessment of sea cucumber populations from the Aegean Sea (Turkey): First insights to sustainable management of new fisheries. Ocean Coast. Manag..

[17] González-Wangüemert M., Valente S., Aydin M. (2014). Effects of fishery protection on biometry and genetic structure of two target sea cucumber species from the Mediterranean Sea. Hydrobiologia.

[18] Gaudron S.M., Kohler S.A., Conand C. (2008). Reproduction of the sea cucumber *Holothuria leucospilota* in the Western Indian Ocean: Biological and ecological aspects. Invertebr. Reprod. Dev..

[19] Dissanayake D.C.T., Stefansson G. (2010). Reproductive biology of the commercial sea cucumber *Holothuria atra* (Holothuroidea: Aspidochirotida) in the northwestern coastal waters of Sri Lanka. Invertebr. Reprod. Dev..

[20] Omar H.A., Abdel Razek F.A., Abdel Rahman S.H. (2013). Reproductive periodicity of sea cucumber *Bohadschia vitiensis* (Echinodermata: Holothuroidea) in Hurghada area, Red Sea, Egypt. Egypt. J. Aquat. Res..

[21] Kubota T., Tomari M. (1998). Reproduction in the Apodid Sea Cucumber Polycheira Rufescens: Semilunar Spawning Rhythm and Sex Change. J. Mar. Biol. Assoc. United Kingd..

[22] Mercier A., Ycaza R., Hamel J. (2007). Long-term study of gamete release in a broadcast-spawning holothurian: Predictable lunar and diel periodicities. Mar. Ecol. Prog. Ser..

[23] Hamel J.-F., Himmelman J.H., Dufresne L. (1993). Gametogenesis and Spawning of the Sea Cucumber *Psolus fabricii* (Duben and Koren). Biol. Bull..

[24] Wigham B., Hudson I.R., Billett D.S.M., Wolff G.A. (2003). Is long-term change in the abyssal Northeast Atlantic driven by qualitative changes in export flux? Evidence from selective feeding in deep-sea holothurians. Prog. Oceanogr..

[25] Hamel J.-F., Mercier A. (1996). Early development, settlement, growth, and spatial distribution of the sea cucumber *Cucumaria frondosa* (Echinodermata: Holothuroidea). Can. J. Fish. Aquat. Sci..

[26] Marquet N., Conand C., Power D.M., Canário A.V.M., González-Wangüemert M. (2017). Sea cucumbers, *Holothuria arguinensis* and *H. mammata*, from the southern Iberian Peninsula: Variation in reproductive activity between populations from different habitats. Fish. Res..

[27] Santos R., Dias S., Tecelão C., Pedrosa R., Pombo A. (2017). Reproductive biological characteristics and fatty acid profile of *Holo-thuria mammata* (Grube, 1840). SPC Beche-De-Mer Inf. Bull..

[28] Azevedo e Silva F., Brito A.C., Simões T., Pombo A., Marques T.A., Rocha C., Sousa J., Venâncio E., Félix P.M. (2021). Allometric relationships to assess ontogenetic adaptative changes in three NE Atlantic commercial sea cucumbers (Echinodermata, Holothuroidea). Aquat. Ecol..

[29] Benítez-Villalobos F., Avila-Poveda O.H., Gutiérrez-Méndez I.S. (2013). Reproductive biology of *Holothuria fuscocinerea* (Echinodermata: Holothuroidea) from Oaxaca, Mexico. Sex. Early Dev. Aquat. Org..

[30] Despalatović M., Grubelić I., Šimunović A., Antolić B., Žuljević A. (2004). Reproductive biology of the holothurian *Holothuria tubulosa* (Echinodermata) in the Adriatic Sea. J. Mar. Biol. Assoc. United Kingd..

[31] Kazanidis G., Lolas A., Vafidis D. (2014). Reproductive cycle of the traditionally exploited sea cucumber *Holothuria tubulosa* (Holothuroidea: Aspidochirotida) in Pagasitikos Gulf, western Aegean Sea, Greece. Turk. J. Zoӧl..

[32] Sousa J., Félix P.M., Brito A.C., Venâncio E., Azevedo e Silva F., Simões T., Raposo A., Neves M., Narciso L., Melo R. (2021). The effects of stocking density on physiological traits in Holothuria forskali broodstock. Aquac. Res..

[33] Chernick M.R., Liu C.Y. (2002). The Saw-Toothed Behavior of Power Versus Sample Size and Software Solutions. Am. Stat..

[34] Engels W.R. (2009). Exact Tests for Hardy-Weinberg Proportions. Genetics.

[35] R Development Core Team (2013). R: A Language and Environment for Statistical Computing.

[36] Torrejon-Magallanes J. Package “sizeMat”.

[37] Shiell G.R., Uthicke S. (2006). Reproduction of the commercial sea cucumber *Holothuria whitmaei* [Holothuroidea: Aspidochirotida] in the Indian and Pacific Ocean regions of Australia. Mar. Biol..

[38] Muthiga N.A. (2006). The reproductive biology of a new species of sea cucumber, *Holothuria* (Mertensiothuria) arenacava in a Kenyan marine protected area: The possible role of light and temperature on gametogenesis and spawning. Mar. Biol..

[39] Muthiga N.A., Kawaka J.A., Ndirangu S. (2009). The timing and reproductive output of the commercial sea cucumber *Holothuria scabra* on the Kenyan coast. Estuar. Coast. Shelf Sci..

[40] Ramos-Miranda J., del Rio Rodriguez R.E., Flores-Hernández D., Rojas-González R.I., Gomez-Solano M.I., Cu-Escamilla A.D., Gómez-Criollo F., Sosa-Lopez A., Torres-Rojas Y.E., Juárez-Camargo P. (2017). Reproductive cycle of the sea cucumber *Holothuria floridana* in the littorals of Campeche, Mexico. Fish. Sci..

[41] Félix P.M., Pombo A., Azevedo e Silva F., Simões T., Marques T.A., Melo R., Rocha C., Sousa J., Venâncio E., Costa J.L. (2021). Modelling the Distribution of a Commercial NE-Atlantic Sea Cucumber, Holothuria mammata: Demographic and Abundance Spatio-Temporal Patterns. Front. Mar. Sci..

[42] Tuwo A., Conand C. (1992). Reproductive biology of the holothurian *Holothuria forskali* (Echinodermata). J. Mar. Biol. Assoc. United Kingd..

[43] Conand C. (1993). Reproductive biology of the holothurians from the major communities of the New Caledonian Lagoon. Mar. Biol..

[44] Navarro P.G., García-Sanz S., Tuya F. (2011). Reproductive biology of the sea cucumber *Holothuria sanctori* (Echinodermata: Holothuroidea). Sci. Mar..

[45] Ramofafia C., Byrne M., Battaglene C. (2003). Reproduction of the commercial sea cucumber *Holothuria scabra* (Echinodermata: Holothuroidea) in the Solomon Islands. Mar. Biol..

[46] Ramofafia C., Battaglene S.C., Bell J.D., Byrne M. (2000). Reproductive biology of the commercial sea cucumber *Holothuria fuscogilva* in the Solomon Islands. Mar. Biol..

[47] Foglietta L.M., Camejo M.I., Gallardo L., Herrera F.C. (2004). A maturity index for holothurians exhibiting asynchronous development of gonad tubules. J. Exp. Mar. Biol. Ecol..

[48] Ramofafia C., Byrne M., Battaglene S. (2001). Reproductive biology of the intertidal sea cucumber *Actinopyga mauritiana* in the Solomon Islands. J. Mar. Biol. Assoc. United Kingd..

[49] Leite-Castro L.V., Junior J.D.S., Salmito-Vanderley C.S.B., Nunes J.F., Hamel J.-F., Mercier A. (2016). Reproductive biology of the sea cucumber Holothuria grisea in Brazil: Importance of social and environmental factors in breeding coordination. Mar. Biol..

[50] Boidron-Métairon I.F., McEdward L. (1995). Larval Nutrition. Ecology of Marine Invertebrate Larvae.

[51] Starr M., Himmelman J.H., Therriault J.-C. (1990). Direct Coupling of Marine Invertebrate Spawning with Phytoplankton Blooms. Science.

[52] Watson G.J., Bentley M.G., Gaudron S.M., Hardege J.D. (2003). The role of chemical signals in the spawning induction of polychaete worms and other marine invertebrates. J. Exp. Mar. Biol. Ecol..

[53] Domínguez-Godino J.A., González-Wangüemert M. (2018). Breeding and larval development of *Holothuria mammata*, a new target species for aquaculture. Aquac. Res..

[54] Conand C. (1981). Sexual cycle of three commercially important *Holothurian* species (Echinodermata) from the Lagoon of New Caledonia. Bull. Mar. Sci..

[55] Abdel-Razek F.A., Abdel-Rahmen S.H., El-Shimy N.A., Omar H.A. (2005). Reproductive biology of the tropical sea cucumber *Holothuria atra* (Echinodermata:Holothuroidea) in the Red Sea coast of Egypt. Egypt. J. Aquat. Res..

[56] Hasan M.H. (2005). Destruction of a *Holothuria scabra* population by overfishing at Abu Rhamada Island in the Red Sea. Mar. Environ. Res..

[57] Hashemi A., Taghavimotlagh S.A., Aminrad T., Gaudron S. (2020). Length at First Maturity and Spawning Time of *Holothuria Leu-cospilota* (Brandt, 1835) in the Northern Waters of the Oman Sea (Sistan and Baluchestan Province). J. Mar. Biol..

[58] Acosta E.J., Rodríguez-Forero A., Werding B., Kunzmann A. (2021). Ecological and reproductive characteristics of holothuroids *Isostichopus badionotus* and *Isostichopus* sp. in Colombia. PLoS ONE.

[59] Liu O.R., Thomas L.R., Clemence M., Fujita R., Kritzer J.P., McDonald G., Szuwalski C. (2016). An Evaluation of Harvest Control Methods for Fishery Management. Rev. Fish. Sci. Aquac..

